# Thiopurine S-methyltransferase and Pemphigus Vulgaris: A Phenotype-Genotype Study

**DOI:** 10.30699/ijp.2020.121365.2320

**Published:** 2020-07-16

**Authors:** Maral Mokhtari, Farzaneh Mostanbet, Saideh Nekooee Fard, Golsa Shekarkhar, Mozhdeh Sepaskhah, Maryam Sadat Sadati

**Affiliations:** 1 *Pathology Department, Shahid Faghihi Hospital, Shiraz University of Medical Sciences, Shiraz, Iran*; 2 *Dermatology Department, Shahid Faghihi Hospital, Shiraz University of Medical Sciences, Shiraz, Iran*

**Keywords:** Allele specific PCR, PCR-RFLP, Thiopurine S- methyl transferase, Thiopurine drugs, ELISA test

## Abstract

**Background & Objective:**

Thiopurine drugs are considered as a treatment modality in various autoimmune disorders including pemphigus vulgaris (PV). These drugs are metabolized by an enzyme “Thiopurine S-methyl transferase” (TPMT). Various variants of this enzyme may have decreased activity leading to serious drug side effects. To investigate the phenotype and genotype of TPMT in PV patients receiving thiopurine drugs.

**Methods:**

A total of 50 patients (29 women and 21 men) with pemphigus vulgaris treating with standard dose of Thiopurine drugs were selected. Sex, age, result of liver function test and complete blood count were recorded. Genotyping of two common non-functional allele (TPMT*2 and TPMT*3C) by Allele-specific and RFLP-PCR was performed. TPMT enzymatic level was determined by an ELISA based method.

**Results:**

Of patients, 36 (72%) were found to have normal TPMT level; and 12, (24%) had higher level of enzyme and 2, 4% had low TPMT enzyme, but none of the patients showed mutant TPMT*2 and TPMT*3C alleles. None of the patients showed hepatotoxicity and bone marrow suppression.

**Conclusion:**

The phenotypic assay based on ELISA method may have false positive and misleading results but genotyping using PCR-RFLP and allele specific PCR is accurate, simple and cost-effective and can be used in patients decided to undergo thiopurine treatment.

## Introduction

Pemphigus vulgaris (PV) disease is a group of skin disorders resulting from autoimmunity against intercellular adhesion molecules or components of the basement membrane in the skin and mucosal surfaces, whose treatment relies on different immunosuppressive agents and account for high morbidity and occasional mortality (1,2,3).

Thiopurine drugs could be served as a therapeutic agent in management of PV patients, although they have related to various toxic adverse effects such as myelosuppression, hepatotoxicity, pancreatitis, and flu-like symptoms. Severe myelosuppression, one of the most severe dose-dependent reactions, is considered to be effectuated by the active metabolite, deoxy-6-thioguanosine 5’triphosphate (6-tGN). The most widely characterized enzyme in the metabolism of thiopurines is thiopurine S- methyltransferase (TPMT), which catalyzes the methylation of thiopurines and metabolites to less active and non-toxic forms of thiopurines drugs. A plethora of studies have shown that lower TPMT enzymatic activity is correlated with higher levels of active drug metabolites and increased thiopurine toxicity. Additionally, it is reported that some genetic polymorphism might be associated with lower TPMT enzymatic activity (4-18).

It has been postulated that roughly 0.3% of the population express low or even no TPMT functioning. Such a population is at the greatest risk of myelosuppression. Moreover, it has been revealed that nearly 15% of the population have intermediate TPMT enzymatic activity with moderate risk of myelosuppression with thiopurine therapy (14-18). It has been suggested that lower initial doses of thiopurine drugs is probably beneficial to patients with either intermediate or low to no TPMT activity (14-18).

Either genetic or an analysis of TPMT enzymatic activity may determine the status of TPMT. The former involves detection of variant alleles of TPMT enzymes leading to decrease enzymatic activity, whereas the latter can directly specify the function of the TPMT enzyme (4-22).

Routine blood specimens could be served for genotyping and phenotyping assays, as white and red blood cells harbor the necessary genetic material and source of TPMT enzyme, respectively (8, 23). There are some inactivating alleles, i.e. (TPMT*2, TPMT*3A, TPMT*3B, and TPMT*3 C) explain 80% to 95% of individuals with lower TPMT activity. TPMT*2 allele (c.238G>C) causes approximately 100-fold decrease in TPMT activity and very low levels of immunological protein. TPMT*3A allele (c.460G>A and c.719A>G) causes no detectable enzyme activity with around 400- fold decrease in protein levels but TPMT*3B allele (c.460G>A) and TPMT*3C allele (c.719A>G) cause a fourfold and 1.4 fold reduction in protein levels, respectively (6,7,10,16-28).

In this study we evaluated the genotype and phenotype of TPMT enzyme in a subset of pemphigus patients who were receiving thiopurine drugs.

##  Materials and Methods

This research was pre-approved by the ethical committee of Shiraz University Medical Sciences. This study included 50 patients with pemphigus vulgaris disease who were receiving thiopurine drugs and referred to dermatology ward or clinic of Shahid Faghihi hospital, affiliated to Shiraz University of Medical Sciences, between 2018 and 2019.

After taking informed consent, age, sex and results of complete blood count (CBC) and liver function tests (LFT) for investigating possible side effects were recorded.

For evaluation of phenotyping of TMPT enzyme, 5 milliliter blood clot was taken and after separation of serum from cells, the serum was kept in -20 degree centigrade until time of analysis. TPMT enzyme activity was evaluated by an ELIZA method (Shanghai Crystal Day Biotech Co, China) based on kit protocol. The kit uses a double-antibody sandwich enzyme-linked immunosorbent assay (ELISA) to assay the level of Human TPMT in samples. 

For genotyping of TPMT enzyme, two milliliter peripheral blood in ethylene di amine tetra acetic acid (EDTA) (0.5 mM) was obtained and refrigerated for TPMT PCR testing. 

Selecting TPMT*3C and TPMT*2 Alleles:

By considering the location of TPMT*3 alleles, TPMT*3B c.460G>A and the TPMT*3C c.719A>G variants are positioned in cis arrangement to the TPMT*3A allele, so, detection of the TPMT: NM_000367.4:c.719A>G (TPMT*3C, dbSNP: rs1142345 G allele) variant alone will also result in all TPMT*3A variants detection (which may need to be further classified by TPMT*3B genotyping) (27).

Due to the mentioned reasons and previous studies in Iranian population showing TPMT*2 and TPMT*3C as the most common variants of TPMT, we selected TPMT*3C and TPMT*2 variants for genotyping using RFLP-PCR and ARMS PCR methods, respectively (14, 20, 21, 26).

PCR-Restriction Fragment Length Polymorphism (PCR-RFLP) Analysis for Genotyping of TPMT c.719A>G (TPMT*3C):

DNA from peripheral blood was extracted using the blood Genomic DNA extraction, Mini kit (50prep) (FAVORGEN) Taiwan, following the manufacturer’s instructions. The extracted genomic DNA was stored at -20ºC until analysis. Two primers PCP-0027 (5’-CACCCAGCCAATTTTGAGTA-3’) and PCP-0028 (5’- CAGGTAACACATGCTGATTGG-3’), with identical thermo-cycling conditions were used for genotyping. PCR protocol was as below: initial denaturation at 95ºC for 10 min, subsequent denaturation at 95ºC for 30 s, annealing for 30 s at 60ºC for 40 cycles, extension at 72ºC for 1 min, and final extension at 72ºC for 10 min.

 The PCR products were electrophoresed in 2.5% agarose, stained with Gel RED and visualized under ultraviolet trans-illumination.

PCR-RFLP genotyping was performed as follows: The target region of the TPMT genes which was amplified by the aforementioned PCR protocol was digested by the AccI restriction enzyme (New England Biolabs, Ipswich, MA, USA). The protocol was as below: 10microL unpurified PCR product, 1 microliter AccI restriction enzyme, 2.5macroL Cut Smart Buffer (New England Biolabs, Ipswich, MA, USA) and 6.5 micro liter nuclease-free water. 

This mix was incubated at 37ºC (for AccI digestion) overnight on a thermal Block (Eppendorf, Hamburg, Germany), and 10 minutes at 80C. Then digested PCR product was electrophoresed in a 3% agarose gel and visualized under UV trans-illumination.

Allele Specific PCR for G238C Mutation (TPMT*2):

To amplify the target region of TPMT*2, two primers were used; DNA: P2C (5' –TAAATA GGA ACC ATC GGA CAC-3') (as reverse primer) and either P2W (5' - GTA TGA TTT TAT GCA GGT TTG-3') or P2M (5' -GTA TGA TTT TAT GCA GGT TTC-3') (as forward primer) for the wild type specific or mutant specific reactions, respectively.

PCR amplification steps were as follows: initial denaturing step at 94ºC for 5 minutes, 35 cycles of denaturing at 94ºC for 30 sec, annealing at 57ºC for 30 seconds, extension at 72ºC for 1 minute and final extension at 72ºC for 5 minutes in an Eppendorf Thermocycler. PCR product was expected to have 254 base pair (bp) length. 

Sanger sequencing was performed for two patients with low enzyme level but normal RFLP- PCR. These two patients had decreased enzymatic level in phenotypic study.

## Results

Fifty PV patients were surveyed in the current study, including 21 men (42%) and 29 women (58%). The mean age of the patients was 46.23±13.26 years. Table 1 shows the laboratory findings of patients.

**Table 1:** Laboratory results of the PV patients receiving thiopurine drugs

Parameter	Mean	SD
AST (U/L)	20.42	9.11
ALT (U/L)	17.20	8.31
Platelet (×1000/µL)	254.46	72.98
Hemoglobin (g/dl)	13.41	1.95
WBC (×1000/µL)	9.21	2.50

ALT: Alanine Aminotransferase; AST: Aspartate Aminotransferase; WBC: white blood cell.

**TMPT Enzyme Level**

In the present study, the average TPMT level of patients was 46.25±55.83 ng/mL. On such basis of normal values offered by ELIZA kit (Normal range：7.8-43.2ng/mL), most patients (36, 72%) were found to have normal TPMT level. Twelve (24%) patients had higher level of enzyme and 2, 4% had low TPMT enzyme. 

In this study, no significant difference was found between patients with normal and abnormal TPMT levels in terms of all laboratory parameters. Table 2 indicates the laboratory parameters between the two groups.

**Table 2:** Comparison of laboratory parameters between patients with normal and abnormal TPMT levels

Parameter	Normal TPMT level (n=36)	Abnormal TPMT level (n=14)	P-value
AST (U/L)	19.91 ± 9.12	21.71 ± 9.29	0.53
ALT (U/L)	16.94 ± 8.57	17.85 ± 7.88	0.73
Platelet (×1000/µL)	255.33 ± 76.96	252.21 ± 64.21	0.89
Hemoglobin (g/dl)	13.51 ± 1.87	13.16 ± 2.21	0.57
WBC (×1000/µL)	9.07 ± 2.36	9.57 ± 2.91	0.53

ALT: Alanine Aminotransferase; AST: Aspartate Aminotransferase; WBC: white blood cell.

**Genotyping of TPMT**

**Fig. 1:**
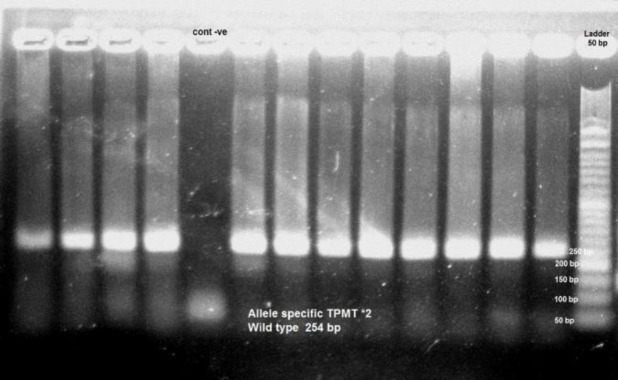
Allele specific TPMT wild type TPMT*2

**Fig. 2:**
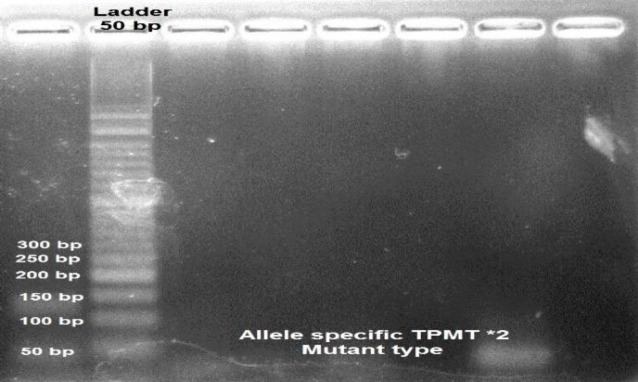
TPMT Allele specific TPMT*2 mutant type

Fifty patients checked for TPMT *2 (analysis of the G238C mutation) with allele specific PCR method. All of them showed 254 bp product compatible with wild type TPMT*2 allele. Figure 1 demonstrates the Allele specific TPMT wild type TPMT*2. None of them showed allele specific mutant TPMT*2 type. Figure 2 demonstrates the TPMT Allele specific TPMT*2 mutant type. 

**Fig. 3:**
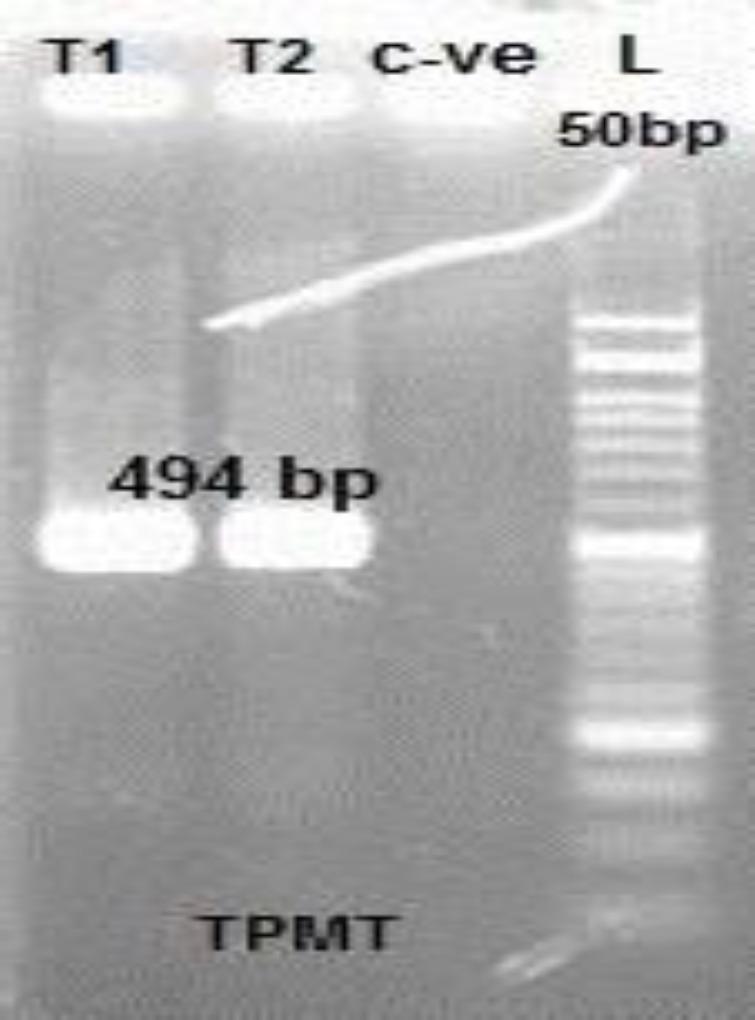
TPMT *3C before adding restriction enzyme (digestion)

Also, PCR-Restriction Fragment Length Polymorphism (PCR-RFLP) Analysis for Genotyping of TPMT c.719A>G (TPMT*3C) was performed in all patients. The PCR results demonstrated that all patients showed a product of 494 bp compatible with wild type TPMT*C before digestion by AccI. Figure 3 demonstrates the TPMT *3C before adding restriction enzyme (digestion).

**Fig. 4:**
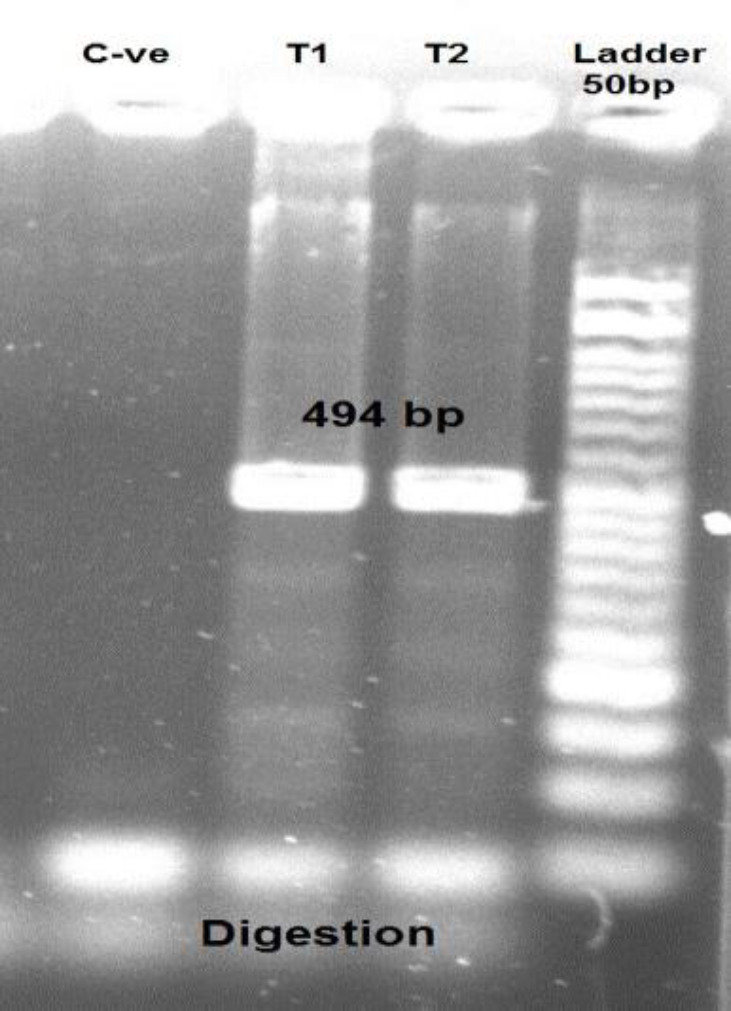
TPMT*C products after adding digestion enzyme

After digestion, the 494 bp product from the wild type TPMT samples remained in all samples, so we did not find any additional bands at about 314 and 180 bp, corresponding to predicted digestion products of the TPMT*3C allele. Figure 4 demonstrates the products after adding digestion enzyme.

We found that in 2 patients the TPMT enzyme level was reduced, but in genotype assay no specific mutant allele was detected. We decided to perform Sanger sequencing for TPMT*C in those two patients. The result of Sanger sequencing also confirmed the result of TPMT-RFLP which showed no mutation. The result of biochemical and LFT was also within the normal limits in these two patients compatible with genotype results.

## Discussion

Thiopurine S-methyltransferase (TPMT) is an active enzyme in the metabolism of thiopurine-based drugs. These thiopurine drugs such as Azathioprine and 6-mercaptopurine are mostly used as steroid-sparing agents in chronic inflammatory conditions, such as PV (4-18). 

At the present time, there is no obvious link between the presence of any inactivating variant TPMT alleles and specific medical disorder. However, it has been suggested that lower TPMT enzyme activity or the presence of a variant allele can increase the risk of thiopurine related drug toxicity. Therefore, it has been recommended to decrease thiopurine starting dose in patients with intermediate or low TPMT activity, while those with absent TPMT activity may benefit from other therapies (14-18).

**TPMT Phenotyping**

Originally a radiochemical method described by Weinshilboum* et al. *has been used for phenotyping assays. But over the years this method has been replaced by a nonradioactive detection method by high performance liquid chromatography (HPLC) (8). TPMT enzyme activity could be measured in red blood cells directly or in serum, Although in patients who have received recent blood transfusions or in the patients with leukemia, because of atypical hematopoiesis, the result will not reflect the true enzymatic activity of the patient (8,11,26). Requiring a fresh blood sample is another drawback of RBC phenotypic assay (18).

To overcome this problem, we evaluated the TPMT level by an immunologic method based on Sandwich ELISA which needs the serum sample.

The average TPMT level of patients was 46.25±55.83 ng/ml in the present study, The TPMT enzyme level was found normal in 72% of patients, while lower and hyper TPMT levels were found in 4% and 24% of patients, respectively. 

In the present study, no significant differences were found between patients with normal and abnormal TPMT level in terms of LFT and CBC laboratory parameters. No significant correlation was also found between TPMT level and laboratory parameters.

In the Gisbert study (29), TPMT Activity in Spain was evaluated in different chronic inflammatory and autoimmune disorders. In this study mean TPMT activity was 20.1 ± 6 U/mL. About 0.5% of patients had low levels and 87.6% had high levels TPMT activity. They also found that the differences among mean TPMT values between the studied disorders were statistically significant.

**TPMT Genotyping**

Several studies stated that the TPMT genotype assays are better indicator of TPMT activity than phenotyping assays and they are used for predicting TGN accumulation or treatment outcome (24).

Sequencing is the gold standard method of TPMT genotyping, but the results will be available with a delay because of the limited accessibility to sequencer. Therefore, other genotyping methods such as PCR-RFLP and allele specific PCR have been studied with comparable result to sequencing. By using these cost-effective methods, the screen time would be reduced significantly and PCR instruments and gel apparatus would be the only required instruments. Such methods are also applicable in small clinical laboratories (27).

In this study we performed TPMT*2 and TPMT*3C genotyping assay using PCR-RFLP and allele specific PCR in 50 PV patients treated by standard doses of AZA. According to the recent studies in Iranian population the most common TPMT variants were TPMT*2 and TPMT*3C.

Our results showed no TPMT*2 or TPMT*3C mutant allele and none of the patients developed hepatotoxicity and bone marrow suppression. Using PCR-RFLP method, Oender* et al. *studied about the most prevalent mutant TPMT*3A and TPMT*3C alleles on 871 Caucasian DNA samples. Their results showed that 8.61% and 0.23% of their samples carried the TPMT*3A allele and TPMT*3C allele, respectively (24).

Wai-Ying Fong,* et al. *in 2017 studied TPMT*3C (c.719A>G) mutant allele in 60 patients using PCR-RFLP method and found 98.3% of patients with the wild-type allele (*1/*1), one (1.7%) with heterozygous (*3C/*1) and none with homozygous (*3C/*3C) alleles. Furthermore, they documented 100% concordance between the results of PCR-RFLP compared to the Sanger sequencing results (27).

In another study by Chi-Chun Ho in 2017 the common loss-of-function TPMT*3C c.719A>G was studied using amplification refractory mutation system polymerase chain reaction (ARMS-PCR). They found only one case of heterozygous TPMT*3C c.719A>G (28). They also stated that genotypes results were 100% concordant with Sanger sequencing (28). 

**TPMT Genotype-Phenotype Concordance**

In our research we evaluated an ELISA based phenotype assay for TPMT in our pemphigus vulgaris patients and we demonstrated that two patients showed decreased level of TPMT antigen, but the patient showed normal lab data despite receiving standard doses of AZA.

None of these cases showed mutation in TPMT*C and TPMT* 2 using RFLP –PCR and allele specific PCR and also Sanger sequencing (which was performed in two patients with low TPMT antigen). Therefore, it seems that the phenotypic assay based on ELISA method is associated with false positive results the results may be misleading. Although a very remote possibility is the inheritance of TPMT* 3B mutant allele, since we did not check that mutation due to its rare occurrence.

Several studies have evaluated the phenotype and genotype concordance for determining the TPMT status. It has been stated that the association between TPMT genotypes and phenotypes does not reach 100% (20,23). 

This means that genetic polymorphism is a very important factor resulting in variation of TPMT activity. The enzyme activity depends on some other factors such as gene expression, interacting and environmental factors including chemicals, drugs or diet components. Some factors affecting the TPMT activity includes gender (higher in males), comorbid conditions (renal failure) and co-administration of certain medications. Some drugs such as sulfasalazine reduce and other drugs such as methotrexate and trimethoprim stimulate TPMT activity, in vitro (10,20,30,31). Although the influence of methotrexate on TPMT activity is controversial and some authors found no effect (11).

The general congruence between phenotype and genotype in healthy people equals 98.4% but in the intermediate range of TPMT activities the value becomes 86%, which might be due to novel mutations (18). Wai-Ying Fong,* et al. *(27) showed that the genotype-phenotype concordance was 97% in their study. 

In the study by Mozafari S* et al. *(26) in Tehran University in 2017, phenotyping by HPLC and genotyping by ARMS-PCR and RFLP-PCR were performed. Samples obtained from 73 healthy adults and 10 children with acute lymphoblastic leukemia. They showed that the HPLC method was accurately used in routine clinical laboratory tests. A normal TPMT activity was detected in 82.19 % of volunteers and the frequency of wild type TPMT alleles was 97.95%. In this study the association between TPMT phenotypes and genotypes was 84.93%.

## Conclusion

In conclusion, the phenotypic assay based on ELISA method may be associated with false positive results, but genotyping using PCR-RFLP and allele specific PCR would be accurate, simple and cost-effective, and can be used in those patients who are candidate to receive AZA treatment. In this study we did not find any mutation in the studied patients but as long as the mutant alleles are recurring with low frequency, another study with larger sample and confirmation by sequencing would be warranted to definitely validate the PCR-RFLP and allele specific methods.
